# Measuring landslide vulnerability status of Chukha, Bhutan using deep learning algorithms

**DOI:** 10.1038/s41598-021-95978-5

**Published:** 2021-08-12

**Authors:** Sunil Saha, Raju Sarkar, Jagabandhu Roy, Tusar Kanti Hembram, Saroj Acharya, Gautam Thapa, Dowchu Drukpa

**Affiliations:** 1 Department of Geography, University of Gour Banga, Malda, West Bengal India; 2 Department of Civil Engineering, Delhi Technological University, Delhi, India; 3 Department of Geography, Nistarini College, Purulia, West Bengal India; 4 National Centre for Hydrology and Meteorology, Thimphu, Bhutan; 5 Royal Government of Bhutan Projects, Phuentsholing, Bhutan; 6 Department of Geology and Mines, Ministry of Economic Affairs, Thimphu, Bhutan

**Keywords:** Environmental social sciences, Natural hazards, Engineering

## Abstract

Landslides are major natural hazards that have a wide impact on human life, property, and natural environment. This study is intended to provide an improved framework for the assessment of landslide vulnerability mapping (LVM) in Chukha Dzongkhags (district) of Bhutan. Both physical (22 nos.) and social (9 nos.) conditioning factors were considered to model vulnerability using deep learning neural network (DLNN), artificial neural network (ANN) and convolution neural network (CNN) approaches. Selection of the factors was conceded by the collinearity test and information gain ratio. Using Google Earth images, official data, and field inquiry a total of 350 (present and historical) landslides were recorded and training and validation sets were prepared following the 70:30 ratio. Nine LVMs were produced i.e. a landslide susceptibility (LS), one social vulnerability (SV) and a relative vulnerability (RLV) map for each model. The performance of the models was evaluated by area under curve (AUC) of receiver operating characteristics (ROC), relative landslide density index (R-index) and different statistical measures. The combined vulnerability map of social and physical factors using CNN (CNN-RLV) had the highest goodness-of-fit and excellent performance (AUC = 0.921, 0.928) followed by DLNN and ANN models. This approach of combined physical and social factors create an appropriate and more accurate LVM that may—support landslide prediction and management.

## Introduction

Among the numerous natural hazards, landslides are considered to be one of the biggest, as they can cause tremendous loss of life and property as well as affect the natural ecosystem. Now attention is being shifted towards the issue of landslides, as increasing developmental works in the Himalayan areas are taking place^1^. The Himalayan region's tectonic fragility is already illustrated, with an overabundance of literature focusing on the broad scale and few on the micro-scale. Being a part of this region, Bhutan has long been known as a place sensitive to natural disasters, such as landslides^2^. Therefore, the need for micro-scale study i.e. province-wise study in Bhutan can be fruitful to act locally viz. the present analysis.

In recent decades, the most suitable way to tackle this hazardous event is the spatial assessment of vulnerability to landslides^3,4^. Assessment of geomorphological, geological, tectonic, climate, vegetation, and land practices may help in identifying the area susceptible and vulnerable to landslides^5,6^. The occurrence of landslides is a natural phenomenon but man-made processes are one of the causes of vulnerability to landslides, which makes it difficult to predict the spatial and temporal occurrence of landslides^7^. In this connection, landslide vulnerability maps (LVMs) may be used as the primary method to classify the high-risk zones which are prone to landslides and also help in identifying the variables responsible for the occurrence of landslides^8^. Presently, with the development of various tools, hardware, and availability of data, it has become easy to produce landslide susceptibility and vulnerability maps^9^.

In spatial landslide modelling, the terms "susceptibility" and "vulnerability" are often used interchangeably; however, "susceptibility" often points to causes intrinsic to physical predisposition (e.g., structural and topographical), while "vulnerability" often corresponds to external influences along with causes intrinsic to physical predisposition (e.g., anthropogenic exposure)^10^. Most of the previous landslide studies have not considered the “vulnerability” aspect rather those have modelled the “susceptibility”. Therefore, another exclusivity of the present work also lies in it. According to the report (May 2010) of XVIth SAARC (The South Asian Association of Regional Cooperation) summit hosted by the Royal Government of Bhutan, several social factors are also responsible for landslides. Among them, landslides caused by road cutting are very common. Landslides are more likely due to intensive deforestation for farmland. The potential human influence could well be the outcome of building roadways and other civil constructions on the slopes which are devoid of planning. Blasting is another addendum to these. The landslide problem is only going to get worse as the world's population grows, putting more strain on natural resources. Therefore, the inclusion of these man-made processes as causative factors to produce a spatial vulnerability map is of utmost importance.

Several studies in the past have considered the role of the geo-environmental factors while few have looked at the socio-economic aspects of landslide events. Based on the arrangement of anthropogenic and physical elements in a region, landslides can be accelerated by different geo-environmental as well as socio-economic factors. With the increasing requirements of the growing population, modification of the land cover for agricultural expansion, road and civil construction, and tourism has influenced the stability of slopes. Therefore, an effort has been made to analyze the role of both physical and social aspects for spatial vulnerability mapping of landslides in this study^1^.

Integration of RS-GIS techniques with knowledge-driven methods or data-mining methods in the landslide susceptibility as well as vulnerability assessment using spatial and non-spatial data has been widely used^11^^.^ The knowledge-driven multi-criteria decision approaches (MCDM) and machine learning models (ML) include frequency ratio, evidential belief function, logistic regression, weight-of-evidence, fuzzy logic, artificial neural network, support vector machine, random forest, logistic model tree, boosted regression tree, etc., which are very widely used in modelling the landslide susceptibility rather than landslide vulnerability^11,12^. In most cases, the ML models provided superior results compared to the traditional approaches because the non-linear data can be adequately treated by the ML models with various scales^13^. Numerous works have reported that the ensemble of ML and conventional statistical models provided superior results compared to the single ML model for modelling landslides^14,15^.

Along with ML models, recent works have acknowledged deep learning models i.e. convolution neural network (CNN), deep learning neural network (DLNN), recurrent neural network (RNN), etc. as emergent and more powerful tools for spatial modelling because of better results than the conventional ML models^16,17^^.^ DLNN shows many topologies as they appear with more than one hidden layer than that of the single-hidden-layer neural network. It is used for the extraction, transformation, pattern recognition, and classification tasks of supervised or unsupervised features^17^. In the case of CNN model, high detection accuracy for landslide detection was achieved by Yu et al.^18^. Ghorbanzadeh et al.^19^ on the other hand reported contrasting findings of the CNN approach with three futuristic methods of MLs despite feasibility and high precision. ANN model outperformed different statistical and ML models in identifying landslide-prone areas as reported by Pourghasemi and Rahmati^20^. The deep learning models used in the present study are still not used for vulnerability studies by the researchers. Previously published landslides susceptibility studies on different parts of Bhutan have revealed that none of them applied deep learning models to predict susceptibility or vulnerability.

Based on the above-reviewed literature, in the present study, ANN, CNN, and DLNN have been selected to analyze the landslide vulnerability status of the Chukha district in Bhutan. The risk posed by landslides in the study region, which is located in the southern part of the country, is mainly due to heavy rainfall during monsoon^21^ and weak geology predominantly made of highly fractured and weathered phyllites, slates, and schists with a high quantity of clay minerals^22^. The situation is further aggravated due to anthropogenic activities like road cuttings^23^. The performance of the above said models were evaluated by the most selective validation methods and statistical measures. The pixels-based evaluation of landslide models has been done by the relative landslide density index (R-index) method.

## Materials and methods

### Study area

**Figure 1:**
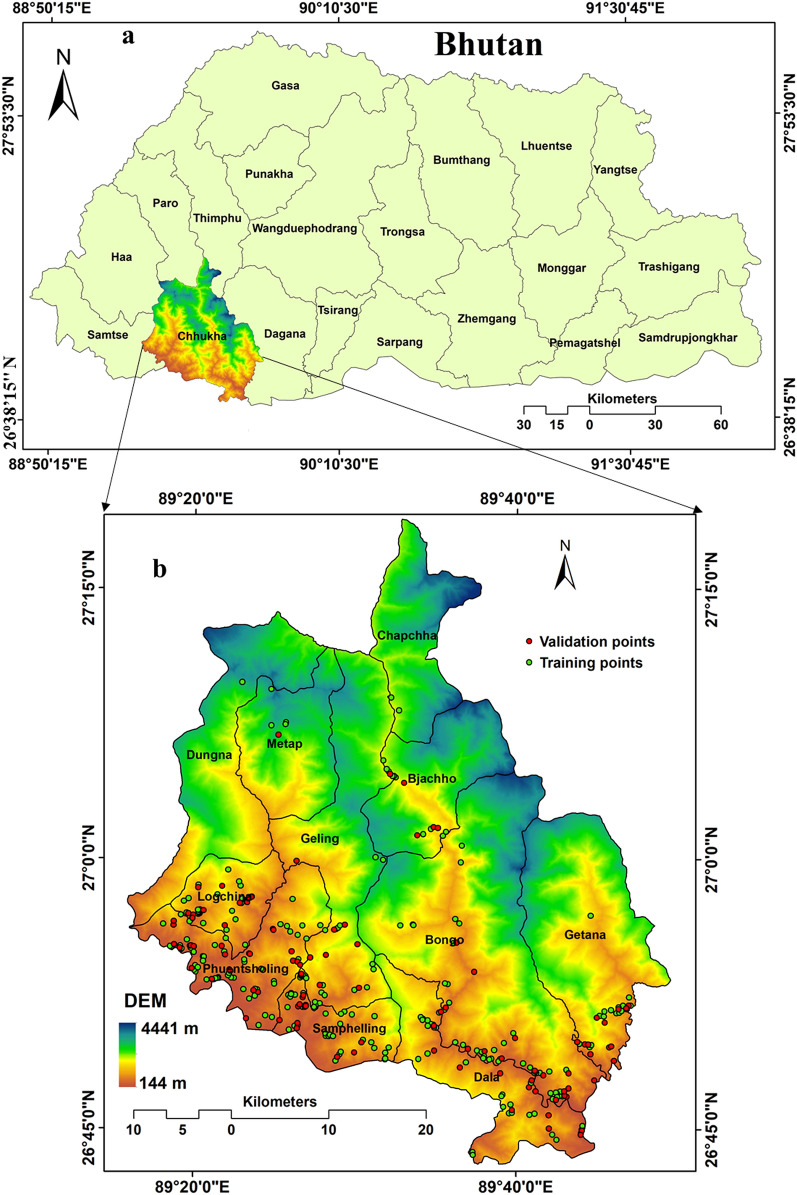
Location of the study area: (**a**) Bhutan and (**b**) Chukha district which is prepared by open source QGIS 3.16 software (https://qgis.org/en/site/forusers/download.html).

The Chukha Dzongkhag (district) lies in the southern foothills of the Bhutan Himalayas (Fig. 1). It shares border with the neighbouring Indian state of West Bengal and thus serves as a vital route for mutual trading between the two countries. The major border town of Phuentsholing is the gateway city that connects India to Western Bhutan. Some of the important hydropower plants such as Chukha and Tala are located in the Chukha district. Because of proximity to India and ease of accessibility to large Indian market, many of the country’s industrial infrastructures are located along the foothill flat areas of Chukha district. From the statistic report of Bhutan Govt., an estimated 4.22% of the entire district (1879.77 km^2^) comprises of fields cultivated with oranges and potatoes that constitutes the main source of rural cash income.

In the study area, the monsoon season extends from June–September of each year and about 78% of the annual rainfall occurs during this period, making the area highly vulnerable to landslide hazards and flooding. In addition, the rugged mountainous terrain with steep slopes further exacerbates the condition that triggers the landslides, exposing the area to the vagaries of weather events. The landslides obstruct the national highway (Phuentsholing-Thimphu Highway) which is the main trade route of the country, causing heavy losses to lives and infrastructures (Table 1). One of the major landslides in the region was along the Phuentsholing-Thimphu highway (26.85 N, 89.33 E to 27.15 N, 89.55 E) just after the 2016 monsoon. The intensity and frequency of such events are expected to increase with climate change, resulting in an increasing risk to the residents and also affecting the economy of the country.

**Table 1:** Details of some recent major landslides and losses occurred in the study area.

Name of the area	Latitude and longitude	Date and time of landslides	Losses caused by the landslides (properties and lives)	Source
Geling gewog (Kamji)	Approx 26.90242 N 89.51761 E	June 16, 2020	Road block	Bhutan Today (National news paper, June 17, 2020)
Sorchen & Kamji	Approx (26.89472 N 89.43556 E)—Sorchen Approx 26.90242 N 89.51761 E—Kamji	July 12, 2019	Road blocks- nearly 200 vehicles stranded	BBS (National TV, July 12, 2019)
Phuentsholing gewog (Kabeytar)	Approx 26.86333 N 89.39611 E	June 25, 2019	Numerous vehicle and houses were damaged	Bhutan 24 × 7 (National newspaper, June 26, 2019)
Phuentsholing gewog (Darjaygang)	Approx 26.8775 N 89.38556 E	August 12, 2017	Killed a 60 year old women and cut off road connection between Dungna and Metedkha gewog	Kuensel (National newspaper, August 13, 2017)
Geling gewog (Kamji)	Approx 26.90242 N 89.51761 E	June 23, 2016	Massive landslide washed away the section of road at Kamji	Kuensel (National newspaper, June 24, 2016)

### Methodology

**Figure 2:**
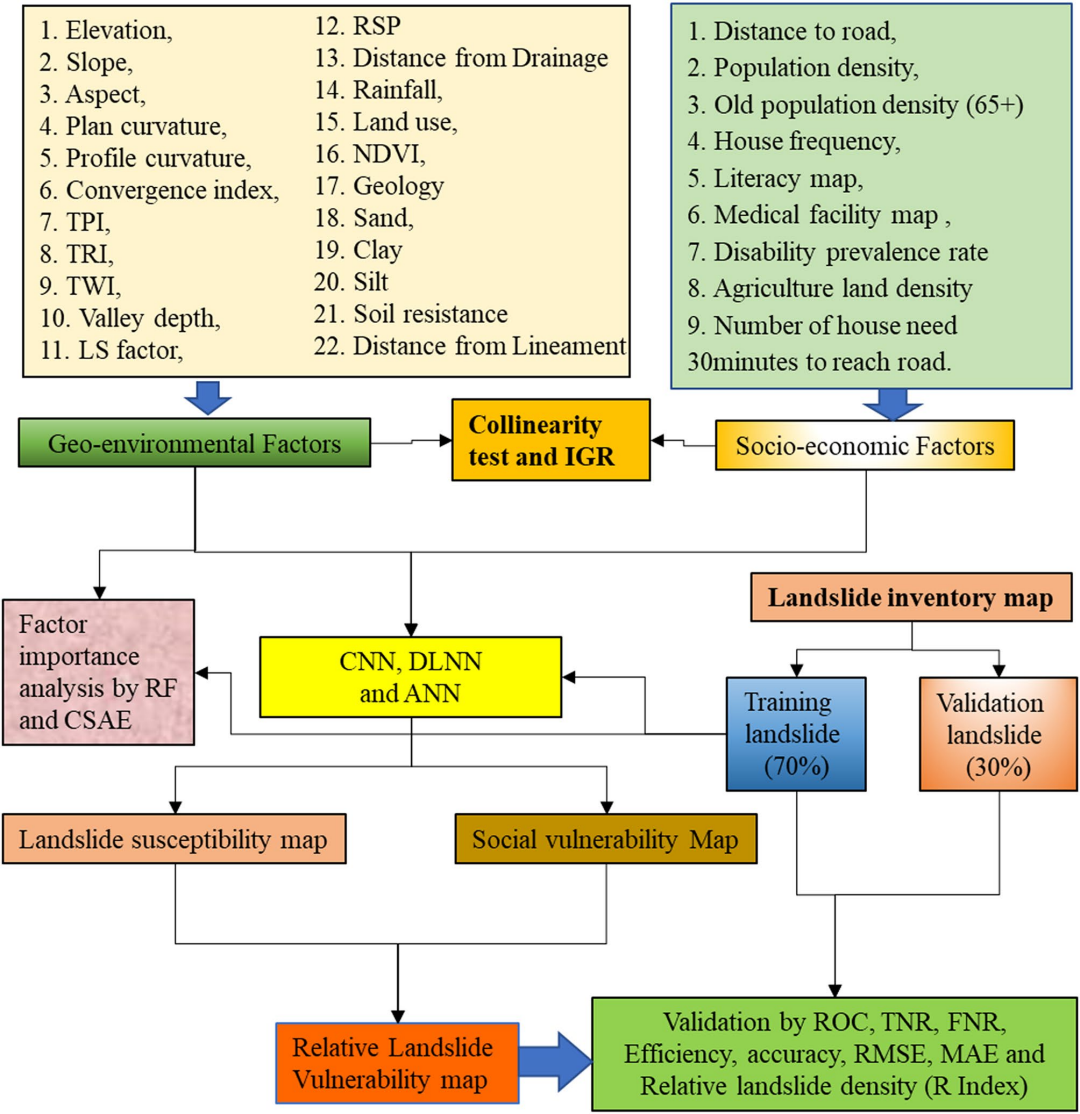
Flowchart of the developed methodology.

The steps followed in this work are presented in Fig. 2.

1. Prepared landslide inventory map based on landslide locations identified through field investigation.
2. Collected data for several geo-environmental and socio-economic factors.
3. Selected factors using collinearity test and information gain ratio (IGR) methods.
4. Applied two deep learning approaches i.e. DLNN and CNN, and one benchmark machine learning model i.e. ANN to produce physical landslide vulnerability, socio-economic landslide vulnerability and overall landslide vulnerability.
5. Analysed significant factors using RF and Chi-square attribute evaluation (CSAE) ML models.
6. Finally, model performances were analyzed using ROC, AUC, efficiency, accuracy, true positive rate (TRP), false positive rate (FPR), true negative rate (TNR), false negative rate (FNR), Kappa index, root mean square error (RMSE), mean absolute error (MAE) and relative landslide density index (R-index) methods.

### Preparation of landslide inventory map (LIM)

**Figure 3:**
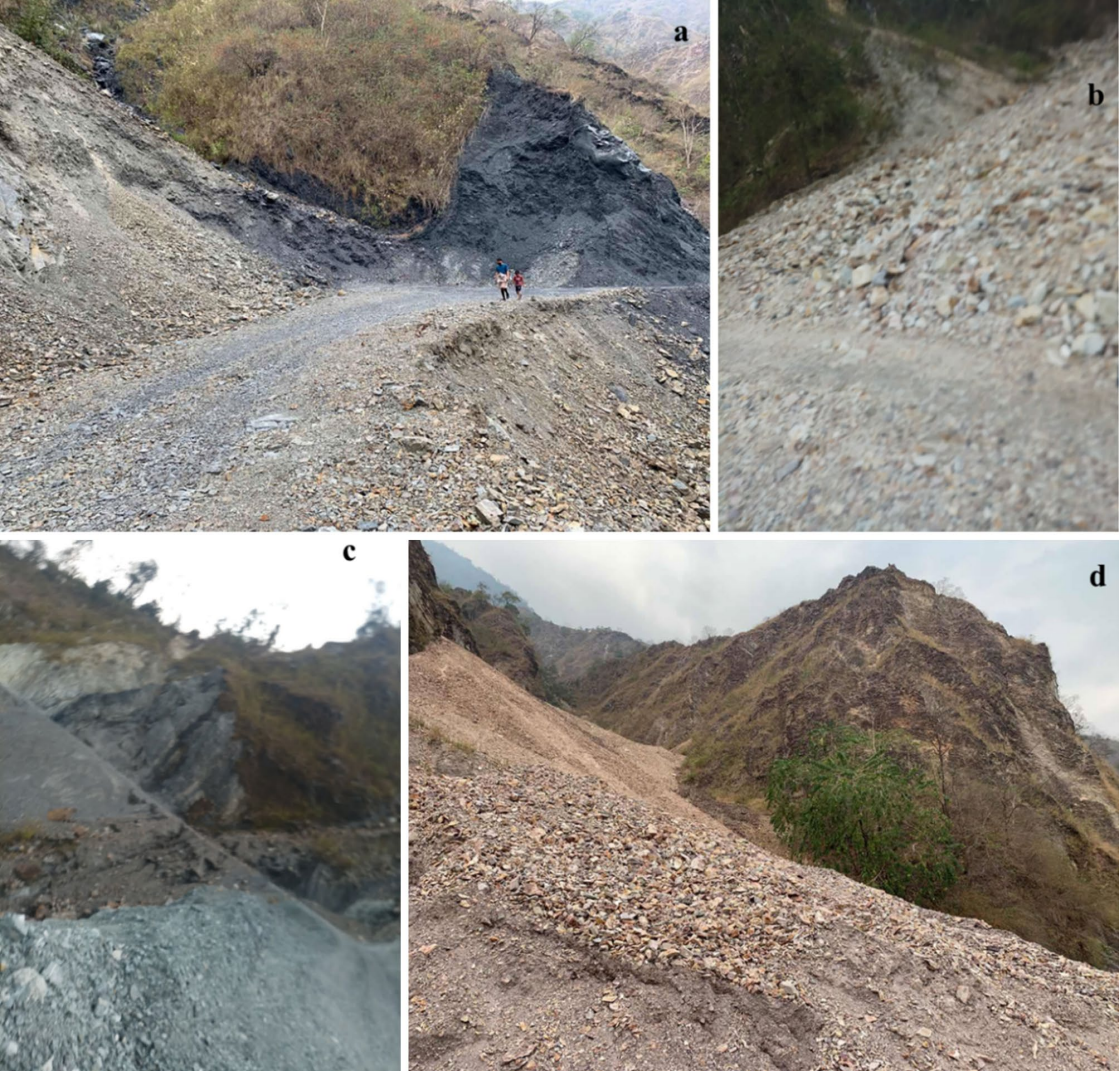
Some field photos of landslide events in Sharphug (latitude 26.76 N, longitude 89.7 E), under Darla Gewog (sub-division) of Chukha Dzongkhag (District).

Mapping of the past and present records of landslide events is called an inventory map^24^. In this study, these records were collected from the Project Dantak, Border Road Organisation (BRO), Govt. of India, and from two Royal Government of Bhutan organizations—Department of Geology and Mines (DGM) and National Centre for Hydrology and Meteorology (NCHM). For location verification, field survey was carried out using Global Positioning System devices in January 2020 (Fig. 3). A total of 350 landslides were mapped for modelling the relative landslide vulnerability, of which 50.4% are rock falls, 40.2% are debris slides and 9.4% are rotational slides. Correspondingly, the same amount of non-landslide points was randomly selected for training and validating the models. Out of the total landslide location, 70% was used as training dataset and the remaining 30% as testing dataset^24^.

### Preparation of the landslide vulnerability conditioning factors (LVCFs)

In this study, 22 geo-environment and 9 socio-economic factors were used for landslide vulnerability modelling which were obtained from the high-resolution (12.5 × 12.5 m) PALSAR DEM of Alaska DEM facility, Landsat 8OLI/TIRS of 30 m × 30 m resolution from USGS Earth Explorer, the demographic data from the National Statistics Bureau, Bhutan, rainfall data of last five years from National Centre for Hydrology and Meteorology, and geological map of 1:500,000 scale from Department of Geology and Mines, Royal Government of Bhutan. The soil resistivity and textural classes (clay, silt and sand) data were extracted from laboratory tests of collected samples from the study area. Data processing and modelling were performed using software such as SPSS, MS-Excel, QGIS 3.16, and R studio.

#### Geo-environmental factors

**Figure 4:**
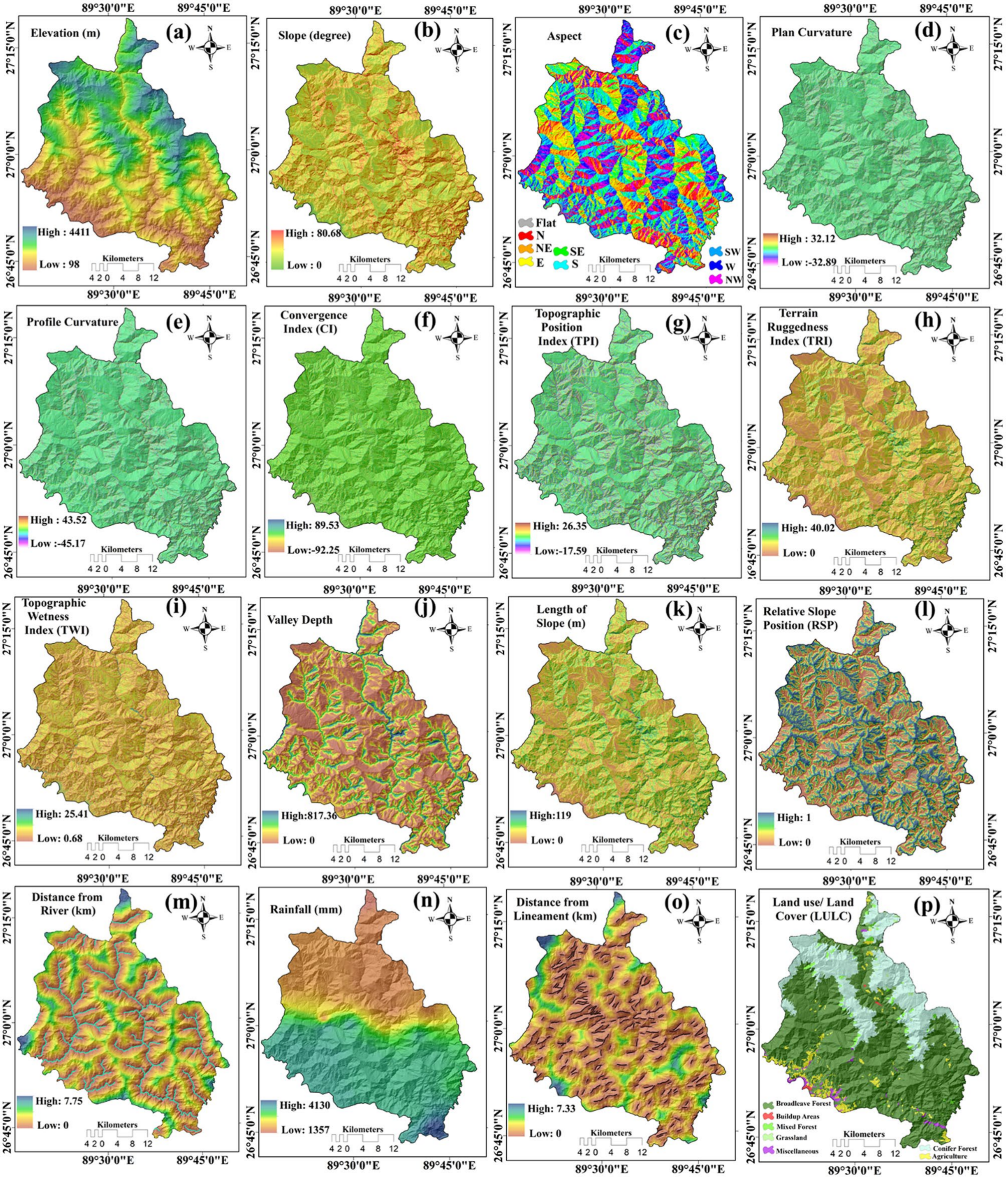

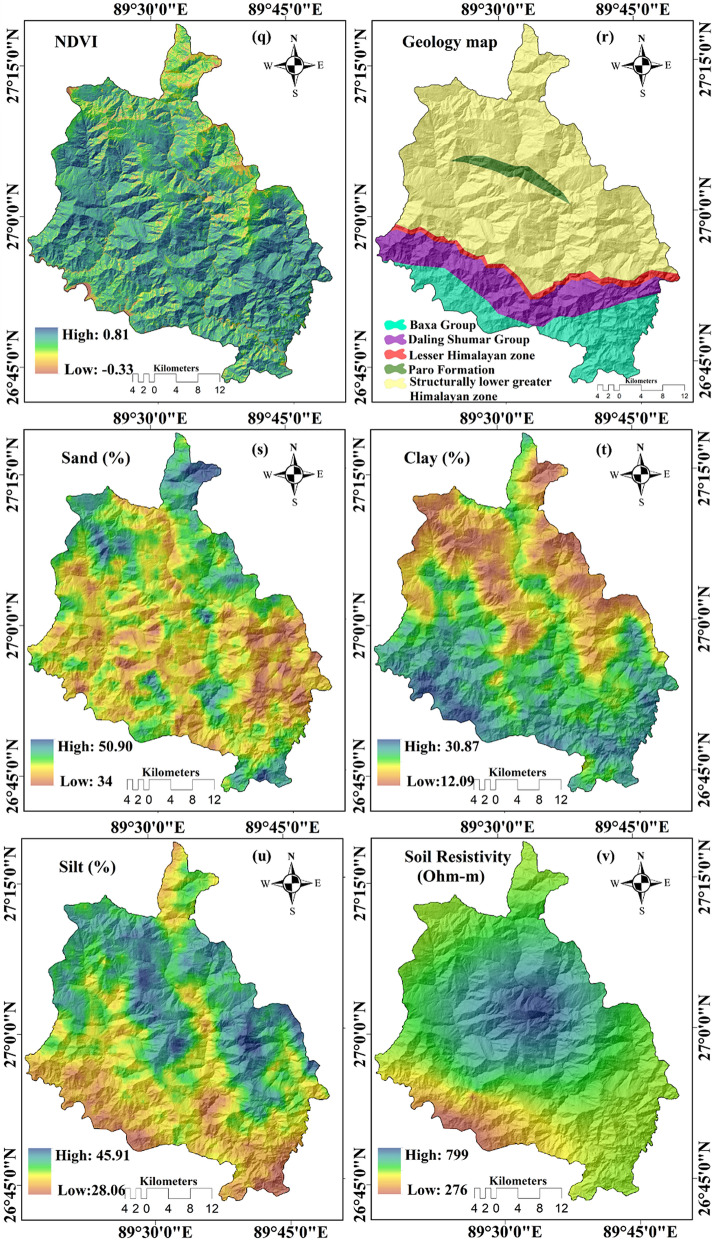
Factors used for producing the landslide susceptibility maps—(**a**) Elevation, (**b**) Slope, (**c**) Aspect, (**d**) Plan curvature, (**e**) Profile curvature, (**f**) Convergence index, (**g**) Topographical position index, (**h**) Terrain ruggedness index, (**i**) Topographical wetness index, (**j**) Valley depth, (**k**) Length of slope, (**l**) Relative slope position, (**m**) Distance from river, (**n**) Rainfall, (**o**) Distance from lineament, (**p**) Land use/land cover, (**q**) Normalized differential vegetation index (NDVI), (**s**) Geology map, (**s**) Sand, (**t**) Clay, (**u**) Silt, (**v**) Soil resistivity.

The elevations of the study area vary from a maximum of 4411 m to as low as 98 m (Fig. 4a) and the slope ranges from 0° to 80° (Fig. 4b). Aspect of slope is classified into nine categories namely, Flat, North, South, West, East, Northeast, Southeast, Northwest, Southwest (Fig. 4c). The spatial distribution of plan curvature and profile curvature ranges from − 32.89 to 32.12 (Fig. 4d) and from − 45.17 to 43.52 (Fig. 4e). The spatial value of convergence index (CI) ranges from − 92.25 to 89.53 (Fig. 4f) and topographical position index varies between − 17.59 and 26.35 (Fig. 4g). The value of terrain ruggedness ranges from 0 to 40 (Fig. 4h). The spatial value of topographical wetness index varies between 0.68 and 25.41 (Fig. 4i) and valley depth varies from 0 to 817 (Fig. 4j). The length of slope and value of relative slope position of this study area ranges between 0 and 119 (Fig. 4k) and from 0 to 1 (Fig. 4l), respectively. Rainfall map was prepared based on the last 5 years' average annual precipitation data of the different stations using the Inverse Distance Weighted interpolation method. The average maximum and minimum precipitations for this study area are 4130 mm and 1357 mm, respectively (Fig. 4n). The land use/land cover (LU/LC) map of the district has been prepared from Landsat 8 OLI/TIRS satellite imagery following the supervised maximum likelihood classification method. The LU/LC categories are broad leave forest, build-up areas, mixed forest, grassland, miscellaneous, conifer forest, and agriculture respectively (Fig. 4p). The normalized differential vegetation index (NDVI) was also generated using RED and NIR band of Landsat 8OLI/TIRS imagery and values differs from − 0.33 to 0.81 (Fig. 4q). The positive values of NDVI indicate the dense vegetation cover areas, while the negative values indicate low vegetation cover areas. The geology of the study can be categorized into Buxa Group, Daling-Shumar Group, the Lesser Himalayan zone, Paro formation, and the structurally lower Greater Himalayan zone (Fig. 4r). The soil resistivity values were determined by surface electrical resistivity method using soil samples and the map depicting resistivity distribution was prepared applying Inverse Distance Weighted (IDW) interpolation method. The soil resistivity spatially varies from 276 to 799 Ω-m (Fig. 4v). Following the same method sand, clay, and silt maps were also prepared. In different parts of the district the percentage of sand, silt, and clay was found to be 34–50%, 12–30%, and 28–45%, respectively (Fig. 4s, 4t, 4u). Drainage of the study area has been extracted from the open series topographical maps and DEM. Buffering tool of QGIS 3.16 was used to prepare the distance from the river map (Fig. 4m) and distance from lineament map (Fig. 4o).

#### Socio-economic factors

**Figure 5:**
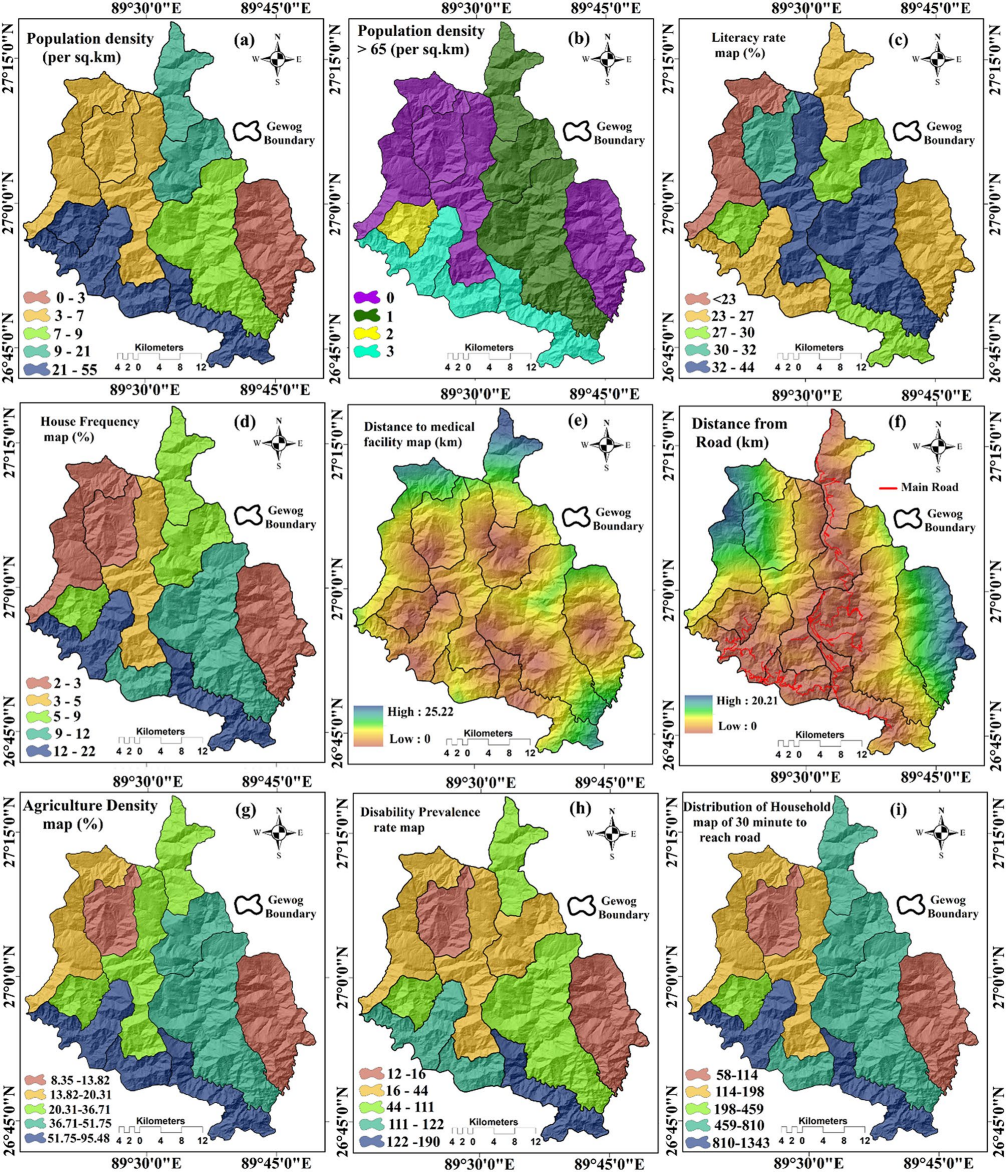
LVCFs used for producing the socio-economic landslide vulnerability maps: (**a**) Population density, (**b**) Old population density, (**c**) Literacy rate, (**d**) House frequency, (**e**) Distance to medical facility, (**f**) Distance to road, (**g**) Agriculture density, (**h**) Disability prevalence rate, (**i**) Household need 30 min to reach road.

To identify the social vulnerability of landslides, socio-economic factors were chosen. The distance from road was prepared using the Euclidian distance buffering tool (Fig. 5f). The demographic data was derived from the district headquarter of Chukha district, Bhutan. The thematic maps of the population density, old population density, house frequency, literacy rate, medical facility, disability prevalence rate, number of households that require at least 30 min to reach the road head (Fig. 5a–i) were prepared in GIS platform based on the block-wise data.

### Factor selection techniques

Collinearity analysis and information gain ratio was used for selecting the appropriate factors to measure the landslide vulnerability of the Chukha district.

#### Collinearity analysis (CA)

Collinearity is a linear association between two explanatory factors. Two factors are perfectly collinear if there is an exact linear relationship between them which can influence the model result. The collinearity test was performed using the Variance Inflation Factor (VIF) and Tolerance (TOI)^25^^.^ A tolerance of less than 0.2 and a VIF of > 5 indicates a multicollinearity problem^26^. Based on the diagnosis, a total of 22 geo-environment and 9 socio-economic factors have been selected to proceed with the modelling.

#### Information gain ratio (IGR) method

The information gain ratio (IGR) is one of the machine learning techniques^27^ which evaluates the correlations of landslide occurrence with landslide conditioning factors and the role of these factors in the frequency of their correlations^28^. The IGR is employed to reduce a bias to multi-value property, taking into account the size and number of sections, when selecting a function. The IGR for element TWI, for example, is estimated as:

1 $$IGR(S,TWI) = \frac{Info(S) - Info(S,TWI)}{{SplitInfo(S,TWI)}}$$

2 $$SplitInfo(S,TWI) = - \sum\nolimits_{j = 1}^{m} {\frac{{\left| {S_{j} } \right|}}{\left| S \right|}} \log_{2} \frac{{\left| {S_{j} } \right|}}{\left| S \right|}$$

where SplitInfo and *S* are represented potential information conducted by partitioning *S* into m subset and training^28^.

## Applied deep learning and benchmark machine learning approaches

### Artificial neural network (ANN)

One of the most popular ANN used for landslide susceptibility is the Multi-layer perceptron Neural Network (MLP-NN)^29^. Basically, three layers which include an input layer, one hidden layer, and an output layer were involved in the topology of MLP-NN models^30^. Although their output is regulated by their structure, the activation functions and the updating of the link weights between the processing components^31^. The MLP-NN was introduced in the current study based on the "RSNNS" R package^32^ and by using a grid search technique, the number of the hidden processing elements was tuned.

### Convolution neural network (CNN)

**Figure 6:**
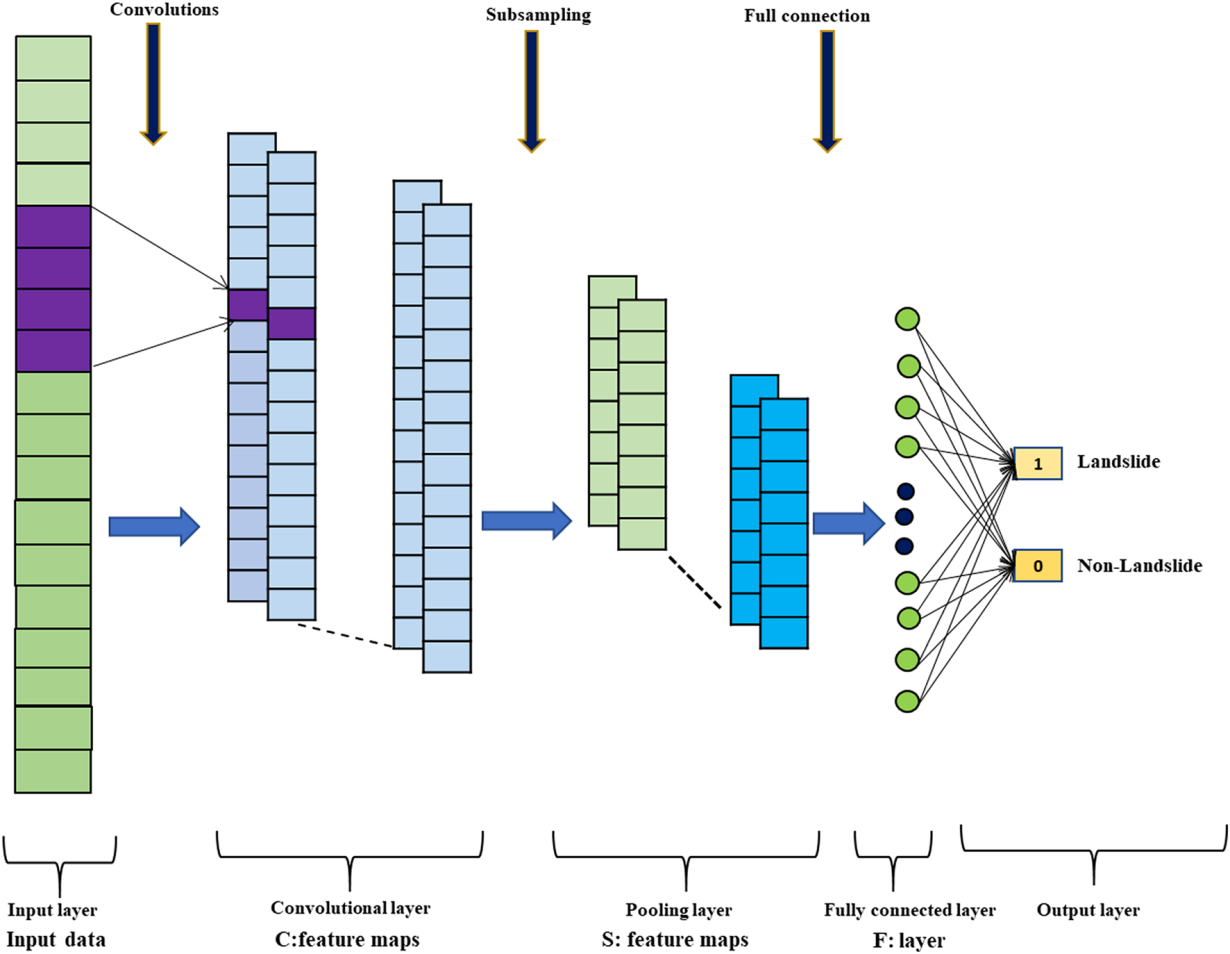
Theoretical structure of used CNN model.

DLAs are modelled on the structure of the human brain and based on an ANN. CNN, introduced by LeCun et al.^33^ is a well-known deep learning algorithms (DLA). Recently, a number of disciplines, including earth science, have been increasingly using CNN for classification and prediction^34^. The utilisation of several layers, pooling, local connections, and mutual weighting distinguishes CNN from traditional neural networks. The central concept behind CNN is that images are used as input parameters. The set of indicators can be greatly decreased and processing can be accelerated. The convolution layers (CLs), pooling layers (PLs), and linear rectified unit layers are involved in the typical structure of CNN model (Fig. 6). CLs deliver the best data classification results by learning the convolutions. By decreasing the quantity of convolution architectures, PLs control overfitting that allows consistent conversion and enhances computational efficiency^35^. The ReLU improves the network's nonlinear capabilities by ReLU activation. Researchers have created and applied numerous analysis structures based on data form, image, and purpose, including GoogleNet^36^, ZFNet^37^, and VGGNet^38^ among the more common structures. Several articles have clarified these layer forms, their basic learning criteria, and how the CNN processes the data for training^33^. The CNN-2D structure is used here because it is relevant for earth science studies^39^. The input data in CNN must be 1-D images: a 1-D input grid cell (vector) with different features which be translated into a 2-D input grid cell to ensure optimum initialization efficiency and this technique is used for mapping landslide vulnerability. In current research, we compare the number of LCVFs to the number of attribute values for each factor, and the larger of the two numbers is used to determine the size of the related two-dimensional matrix. The research region, for example, contains 25 geological categories, which is more than the number of LCVFs. As a result, for each grid cell, we created a 25 × 25 matrix. Since there is no sorting of data and analysis is continuous, the images are large.

### Deep learning neural networks (DLNN)

**Figure 7:**
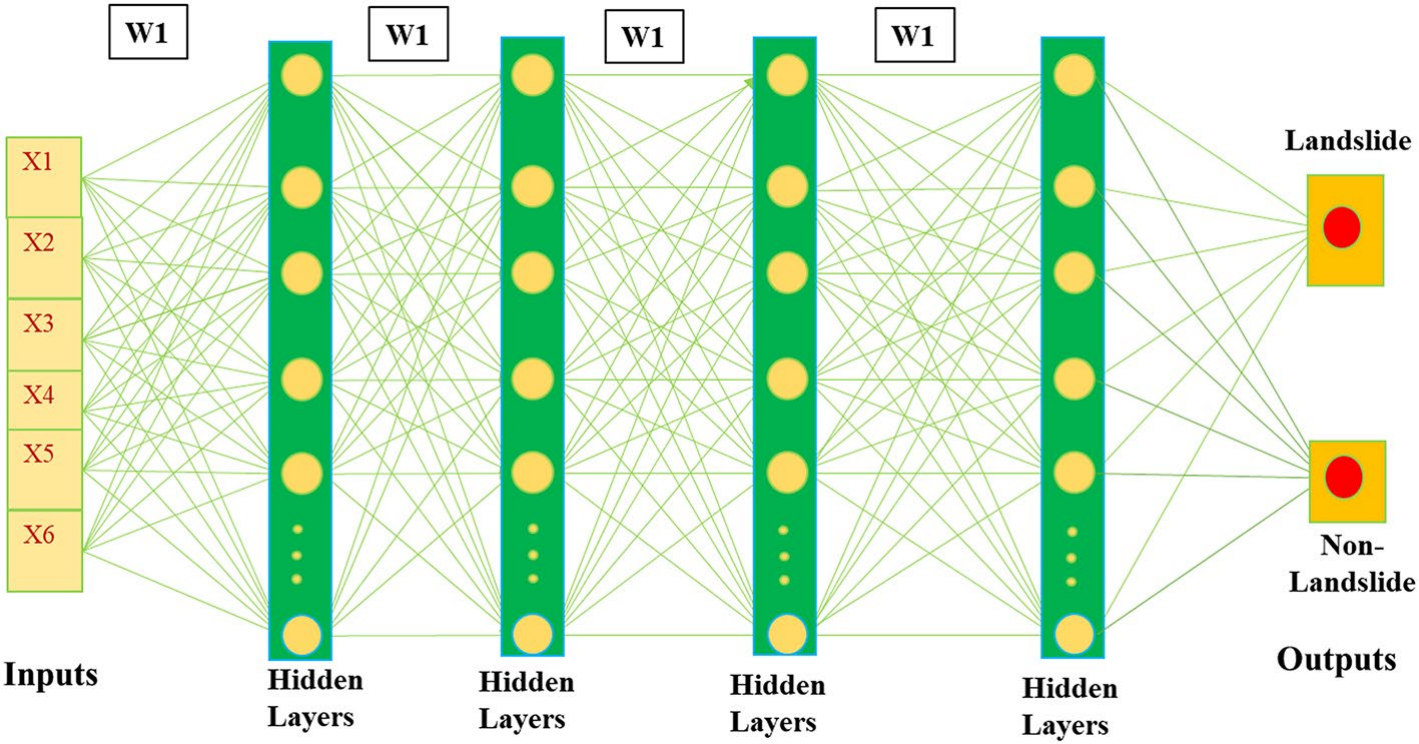
Configuration of the DLNN approach.

The major advantage of DLNN model is that it uses raw dataset to create a high-level function^18^. DLNN comprises of three layers—an input layer, followed by hidden layers which lead to output layers^40^. Figure 7 demonstrates the conceptual setup of the DLNN model included in this analysis. The overall pattern of DLNN model is to work in such a way that the input layer delivers the signals that are diverse landslide factors, and after processing and interpretation of this information in multiple hidden layers, the impacts are shown in the model’s last year, the output layer. The output layer has two probable labels, i.e. a negative label (non-landslides) and a positive label (landslides). From the last hidden layer, these classification results are collected and displayed in the output layer^41^. DLNN has unique advantages over the conventional ML algorithm, and therefore much more attention has been put on the use of the DLNN model in the area of prediction analysis. DLNN outperforms all other ML models in several ways, by making optimum use of unstructured data by specific observations to recognize the training dataset, being versatile enough as to identify new data, and being able to create new learning models by introducing more layers to the neural network architecture.

Mathematical equation mentioned below was applied in DLNN as per the Kim^40^:

3 $$h(x) = \left\{ {\begin{array}{*{20}l} x \hfill & {if\;x > 0} \hfill \\ 0 \hfill & {if\;x < 0} \hfill \\ \end{array} } \right. = \max (0,x)\;h(x) = \left\{ {\begin{array}{*{20}l} x \hfill & {if\;x > 0} \hfill \\ 0 \hfill & {if\;x \le 0} \hfill \\ \end{array} } \right. = \max (0,x)$$

where *x* is an input signal, and *h* is an activation function. The following can be represented on the basis of the ReLU activation function as:

4 $$h(x) = \left\{ {\begin{array}{*{20}l} 1 \hfill & {if\;x > 0} \hfill \\ 0 \hfill & {if\;x \le 0} \hfill \\ \end{array} } \right.$$

The cost function is the distinction between class outcomes that are experiential and expected. The loss function (L) of a cross-entropy is used to detect patterns and is given by:

5 $$L = - \tfrac{1}{{N_{D} }}\sum\limits_{n = 1}^{{N_{D} }} {T{ 1}n(Y) + (1 - T)1n(1 - Y)}$$

where, the number of the training data sets is expressed by $N_{D}$, *T* indicates the class outputs detected and *Y* displays the class outputs expected.

## Methods used for validating the models

### Receiver operating characteristics (ROC) curve

For any two distinct vectors where the first vector describes the binary presence-absence state of a particular action and the second vector gives the related probability predictions, the ROC curve can be prepared which is a common cut-off dependent diagnosis^42,43^. The overall model success can be recognized based on AUC values, as per the classification provided by Hosmer and Lemeshow^44^. There are four major elements of ROC curve: e true positive (P), false positive (Q), false negative (R) and true negative. The following measures including sensitivity or true positive rate (TPR), false positive rate (FPR), true negative rate or specificity (TNR), false negative rate or miss rate (FNR), efficiency, precision, negative predictive value (NPV), Matthews correlation coefficient (MCC) and Cohen’s Kappa have been computed from these elements for validating the models:

6 $${\text{TPR}} = \frac{P}{X} = \frac{P}{P + R}$$

7 $${\text{FPR}} = \frac{Q}{Y} = \frac{Q}{Q + S} = 1 - \frac{S}{S + Q}$$

8 $${\text{TNR}} = \frac{S}{Y} = \frac{S}{S + Q}$$

9 $${\text{FNR }} = \frac{Q}{X} = \frac{Q}{Q + P} = 1 - TPR$$

10 $${\text{Efficiency}}\,\left( {{\text{accuracy}}} \right) = \frac{P + S}{T}$$

where X, Y and T are the number of landslides, non-landslides and sum of total landslides and non-landslides, respectively

11 $${\text{Precision}} = \frac{P}{P + Q}$$

12 $${\text{NPV}} = \frac{S}{S + R}$$

13 $${\text{MCC }} = \frac{{\left( {P \times S} \right) - (Q \times R)}}{{\sqrt {(P + Q)(P + R)(S + Q)(S + R)} }}$$

14 $${\text{Cohen's}}\,{\text{Kappa}} = \frac{{(P + S) - \left[ {\left( {P + R} \right)(P + Q) + (R + S)(Q + S)} \right]/T}}{{T - \left[ {\left( {P \times S)} \right)(P + Q) + (R + S)(Q + S} \right]/T}}$$

### RMSE

The root mean square error (RMSE) was determined on the basis of the variations between the values expected by a model and the values actually observed (Eq. 9):

15 $$RMSE = \sqrt {\frac{{\sum\nolimits_{i = 1}^{N} {\left| {\widetilde{P} - P} \right|^{2} } }}{N}}$$

where N is sample size, $\widetilde{P}$ and *P* is predicted and observed values of dependent variable, respectively.

### MAE

Mean absolute error (MAE) is measured as the sum of the differences between the expected value and the actual value of the model, without taking their direction into account:

16 $$MAE = \frac{1}{N}\sum\limits_{i = 1}^{n} {\left| {\widetilde{P} - P} \right|}$$

where N is sample size, $\widetilde{P}$ and *P* are predicted and observed values of dependent variable, respectively.

### Relative landslide density (R-Index)

R-index was used to evaluate the vulnerability maps of landslides. R-index is calculated as following^45^.

17 $$R = ((xi/Xi)/\sum {(xi/Xi)) \times 100}$$

where *xi* is the percentage of the area that is vulnerable to landslides in each vulnerability class, and *Xi* is the percentage of landslides in each vulnerability class. The maximum values of these vulnerability classes indicate the highest goodness-of-fit and excellent accuracy^46^.

## Results

### Multicollinearity assessment

The findings of collinearity results indicate that no linearity exists among the LVCFs because the Tolerance and VIF values of these factors do not surpass their limits (Table 2) which suggests the aptness for inclusion in the modelling.

**Table 2:** Collinearity results of LVCFs.

LVCFs	Collinearity statistics	
	Tolerance	VIF
Geo-environment factors		
Elevation	0.272	3.812
Slope	0.348	2.706
Aspect	0.898	1.113
Plan curvature	0.390	2.562
Profile curvature	0.282	3.547
Convergence index	0.517	1.933
Topographic position index	0.146	6.844
Terrain ruggedness index	0.270	4.286
Topographic wetness index	0.351	2.848
Valley depth	0.323	3.100
Relative slope position	0.327	3.059
Length of slope	0.181	5.521
Rainfall	0.269	3.723
NDVI	0.778	1.285
Distance from river	0.605	1.653
Socio-economic factors		
Population density	0.665	1.323
Old population density	0.273	3.646
House frequency	0.263	3.808
Literacy rate	0.233	4.286
Distance from road	0.266	3.765
Medical facility	0.627	1.596
Disability prevalence rate	0.123	8.114
Agriculture density	0.232	3.556
Number of the household for 30 min to reach road	0.334	2.269

### Results of IGR

**Figure 8:**
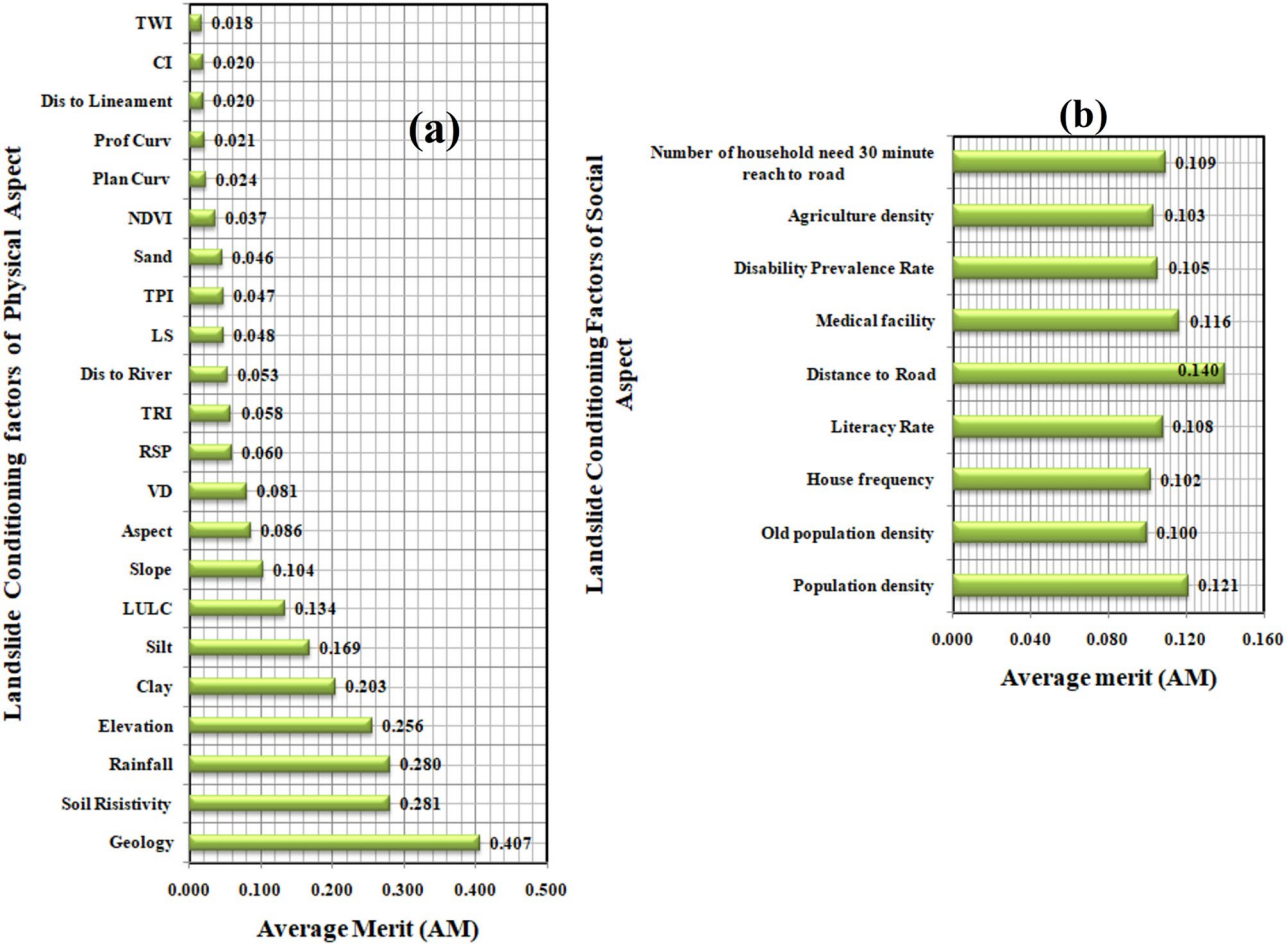
Average merit of LVCFs: (**a**) geo-environment factors, (**b**) socio-economic factors.

The result of IGR method is shown in Fig. 8. The IGR values of the geo-environmental LVCFs for landslide vulnerability prediction were higher for geology, soil resistivity, rainfall, and elevation (Fig. 8a) and for socio-economic LVCFs, it is higher for distance to road and population density (Fig. 8b).

### Landslide vulnerability analysis

**Figure 9:**
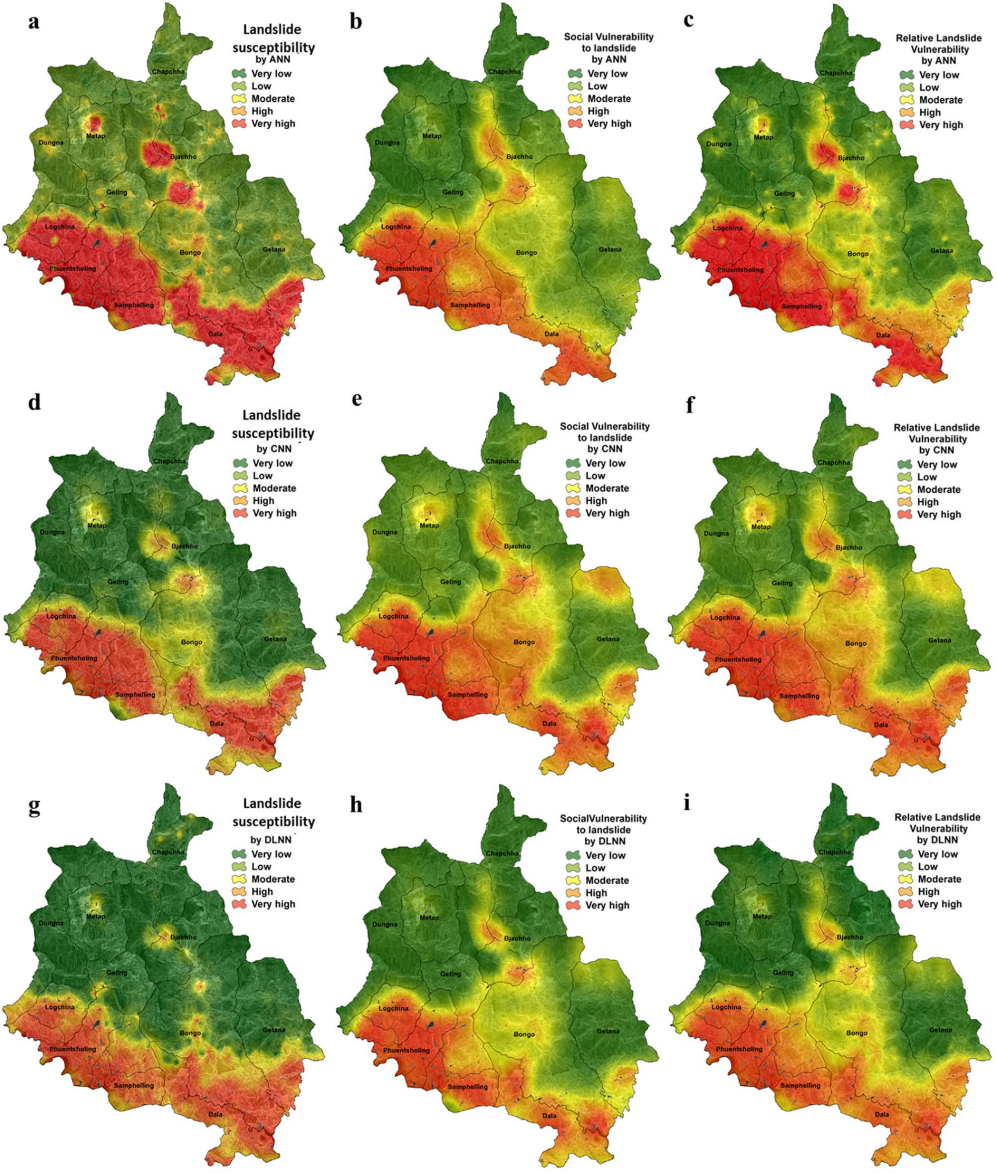
Physical, social and relative landslide vulnerability maps produced by: (**a**) ANN-LS, (**b**) ANN-SV, (**c**) ANN-RLV, (**d**) CNN-LS, (**e**) CNN-SV, (**f**) CNN-RLV, (**g**) DLNN-LS, (**h**) DLNN-SV and (**i**). DLNN-RLV.

**Figure 10:**
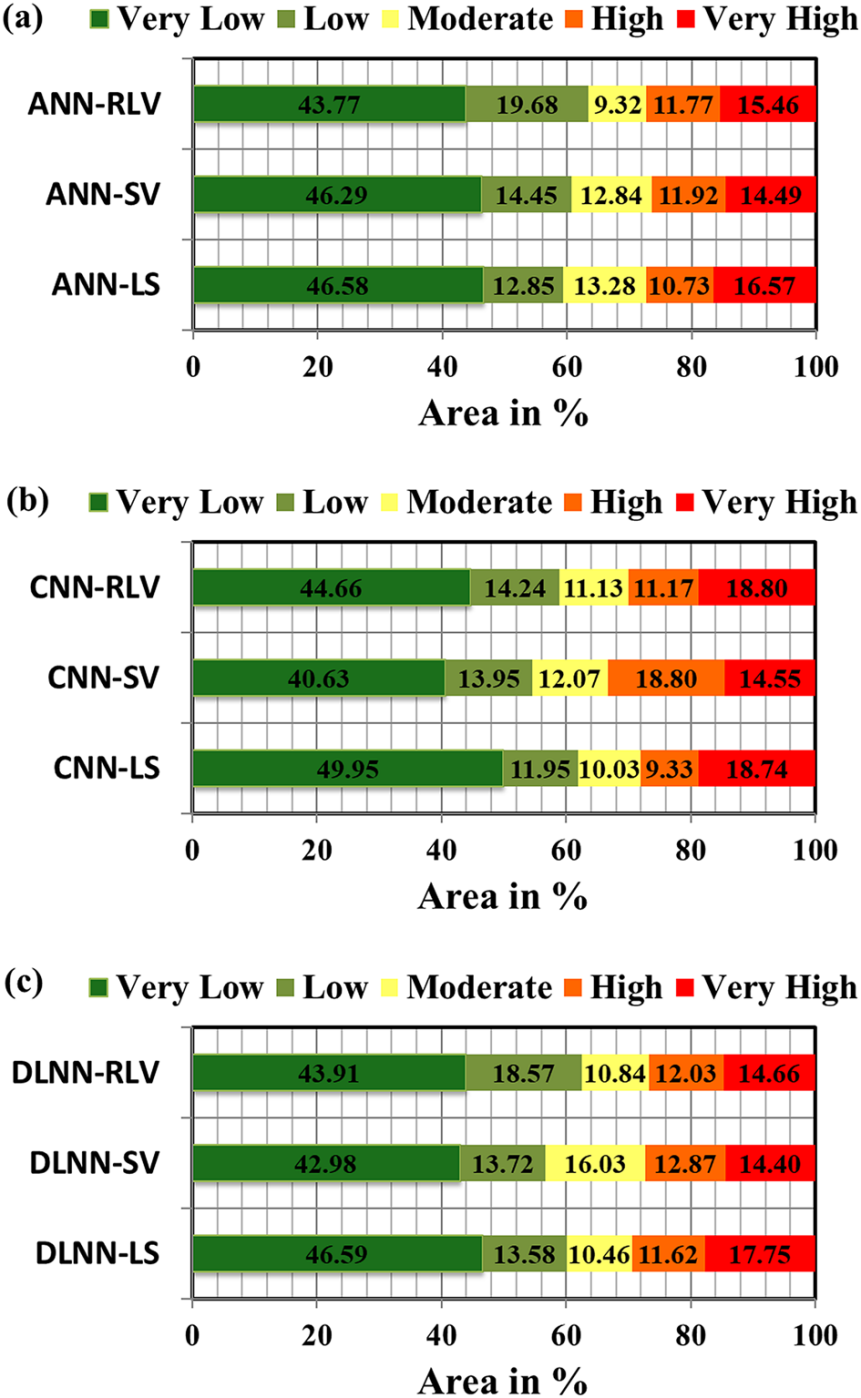
Distribution of physical, social and relative vulnerability classes produced using three models.

Using the three models i.e. ANN, CNN, and DLNN and considering three aspects of vulnerability i.e. geo-environmental, socio-economic, and relative or overall vulnerability, a total of 9 landslide vulnerability maps were derived which are ANN-LS (landslide susceptibility), ANN-SV (social vulnerability), ANN-RLV (relative landslide vulnerability), CNN-LS, CNN-SV, CNN-RLV, DLNN-LS, DLNN-SV, DLNN-RLV. Accepting natural breaks classification method, these landslide susceptibility maps were classified into five vulnerability zones: very low, low, moderate, high, and very high. The ANN-LS, CNN-LS, and DLNN-LS maps have 16.57%, 18.74%, and 17.75% area of the district as very high vulnerable for landslides from the scenario of geo-environmental setup. Similarly, ANN-SV, CNN-SV, and DLNN-SV maps have provided the socio-economic status of vulnerability as very high of 14.49%, 14.55%, and 14.40% area, respectively. In case of relative vulnerability maps ANN-RLV, CNN-RLV and DLNN-RLV have quantified 15.46%, 18.80%, and 14.66% of area as most vulnerable to landslides. The very high vulnerable zones have mostly occurred in the southern, south-west and south-east portion of the district and very low vulnerability is found in northern, north-east, and north-west regions. These results appear to be associated with the presence of weak geology, heavy rainfall during monsoon, and Phuentsholing-Thimphu national highway that passes through the first three portions of the district. Apart from this, rapid population growth in the above areas due to the fast development of Phuentsholing, the business city of Bhutan, resulted in new anthropogenic activities and thus weakening the soil (Fig. 9). The spatial extension of other vulnerability classes is given in Fig. 10.

### Validation

**Figure 11:**
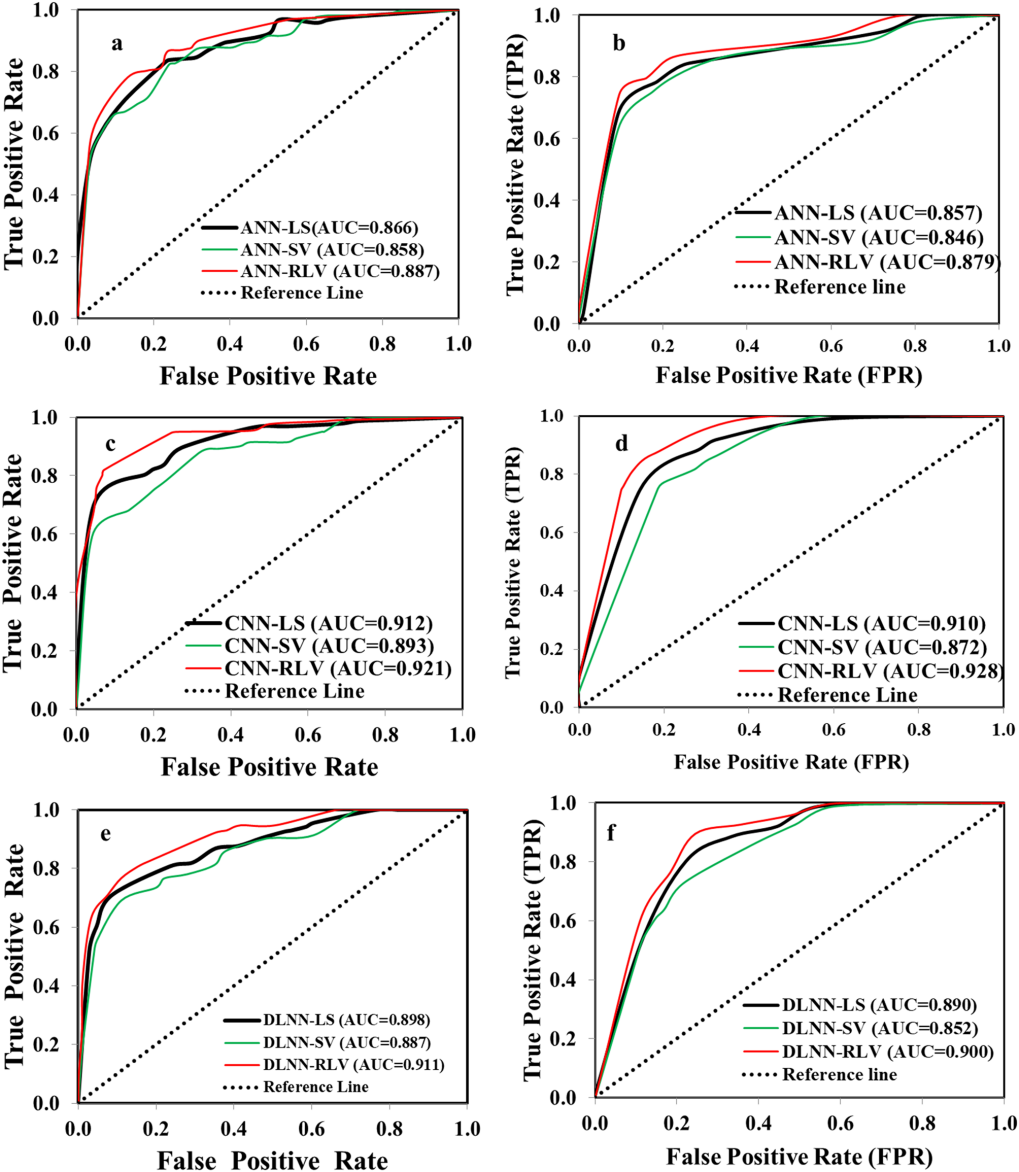
ROC curves used for validation of landslide vulnerability models: (**a**) ANN-LS, ANN-SV and ANN-RLV using Training, (**b**) ANN-LS, ANN-SV and ANN-RLV using validation datasets, (**c**) CNN-LS, CNN-SV and CNN-RLV using Training, (**d**) CNN-LS, CNN-SV and CNN-RLV using validation, (**e**) DLNN-LS, DLNN-SV and DLNN-RLV using training, (**f**) DLNN-LS, DLNN-SV and DLNN-RLV using validation datasets.

Table 3 shows the comprehensive results of the ROC curve and other validation measures. The AUC computed using train data indicates success rate and AUC computed using test or validation data denotes prediction rate of the models^47^. Using the training data, AUC of ANN-LS, ANN-SV and ANN-RLV models are 0.893, 0.864, and 0.907 and using validation dataset these are 0.887, 0.846 and 0.902 respectively. The AUC using train and validation data are 0.912, 0.910 in CNN-LS; 0.893, 0.872 in CNN-SV and 0.921 and 0.928 in CNN-RLV models respectively. The AUC values of DLNN model are 0.880 (DLNN-LS), 0.867 (DLNN-SV) and 0.901 (DLNN-RLV) in the training case and 0.890 (DLNN-LS), 0.852 (DLNN-SV) and 0.900 (DLNN-RLV) in the validation case. Among these models, the CNN-RLV model has achieved the highest performance in terms of the AUC, accuracy, efficiency, kappa and predictive values and all other measures (Table 3). The result of reliability measures such as MAE and RMSE value is lowest for CNN-RLV followed by DLNN-RLV and ANN-RLV model. The R-index values for each model using geo-environmental aspect, socio-economic aspect or overall factors, is highest for very high vulnerability class followed by high vulnerability class (Table 4; Fig. 11).

**Table 3:** Results of validation measures based on training and validation datasets.

Matrices	ANN-PV	ANN-SV	ANN-RLV	CNN-PV	CNN-SV	CNN-RLV	DLNN-PV	DLNN-SV	DLNN-RLV
Training dataset									
TPR	0.81	0.77	0.82	0.84	0.80	0.86	0.83	0.79	0.83
FPR	0.16	0.20	0.14	0.13	0.17	0.10	0.15	0.18	0.13
TNR	0.84	0.80	0.86	0.87	0.83	0.90	0.85	0.82	0.87
FNR	0.19	0.23	0.18	0.16	0.20	0.14	0.17	0.21	0.17
Accuracy	0.82	0.78	0.84	0.85	0.81	0.88	0.84	0.80	0.85
PPV	0.85	0.80	0.86	0.88	0.83	0.90	0.85	0.83	0.87
NPV	0.79	0.76	0.81	0.83	0.79	0.85	0.81	0.77	0.82
MCC	0.65	0.57	0.68	0.70	0.63	0.76	0.69	0.61	0.70
Cohen's Kappa	0.65	0.57	0.68	0.70	0.63	0.75	0.69	0.61	0.70
AUC	0.866	0.858	0.887	0.912	0.893	0.921	0.898	0.887	0.911
MAE	0.150	0.187	0.101	0.032	0.064	0.013	0.040	0.089	0.028
RMSE	0.387	0.432	0.318	0.180	0.254	0.114	0.199	0.299	0.169
Validation dataset									
TPR	0.82	0.82	0.86	**0.89**	**0.86**	0.92	0.88	0.85	0.90
FPR	0.11	0.17	0.10	0.09	0.12	0.07	0.09	0.15	0.09
TNR	0.89	0.83	0.90	0.91	0.88	0.93	0.91	0.85	0.91
FNR	0.18	0.18	0.14	0.11	0.14	0.08	0.12	0.15	0.10
Accuracy	0.85	0.83	0.88	0.90	0.87	0.92	0.90	0.85	0.90
PPV	0.90	0.83	0.90	0.92	0.89	0.93	0.92	0.85	0.90
NPV	0.81	0.82	0.85	0.89	0.86	0.92	0.88	0.85	0.91
MCC	0.71	0.65	0.75	0.81	0.74	0.85	0.79	0.71	0.81
Cohen's Kappa	0.71	0.65	0.75	0.81	0.74	0.85	0.79	0.71	0.81
AUC	0.857	0.846	0.879	0.910	0.872	0.928	0.890	0.852	0.900
MAE	0.069	0.103	0.044	0.023	0.027	0.011	0.024	0.067	0.022
RMSE	0.263	0.321	0.209	0.153	0.163	0.104	0.155	0.259	0.150

**Table 4:** Relative landslide density index of susceptibility classes of LSM models.

Models	Classes	No. of pixels	% of pixels	No. of landslides	% of landslides	R-index
ANN-P	Very low	5,607,364	46.58	21	6.11	2
	Low	1,547,452	12.85	45	12.86	15
	Moderate	1,598,676	13.28	47	13.50	16
	High	1,291,334	10.73	29	8.36	12
	Very high	1,994,494	16.57	207	59.16	55
ANN-S	Very low	5,573,335	46.29	38	10.93	3
	Low	1,739,451	14.45	21	6.11	6
	Moderate	1,546,377	12.84	82	23.47	27
	High	1,435,333	11.92	51	14.47	18
	Very high	1,744,824	14.49	158	45.02	46
ANN-RLV	Very low	5,269,576	43.77	1	0.32	0
	Low	2,369,894	19.68	6	1.61	1
	Moderate	1,121,903	9.32	33	9.32	14
	High	1,416,707	11.77	93	26.69	31
	Very high	1,861,241	15.46	217	62.06	54
CNN-P	Very low	6,013,570	49.95	1	0.32	0
	Low	1,438,915	11.95	8	2.25	3
	Moderate	1,207,156	10.03	20	5.79	9
	High	1,123,336	9.33	60	17.04	28
	Very High	2,256,343	18.74	261	74.60	61
CNN-S	Very low	4,891,668	40.63	2	0.64	0
	Low	1,678,914	13.95	7	1.93	2
	Moderate	1,453,602	12.07	32	9.00	11
	High	2,263,865	18.80	96	27.33	22
	Very high	1,751,272	14.55	214	61.09	64
CNN-RLV	Very low	5,376,679	44.66	1	0.32	0
	Low	1,714,734	14.24	5	1.29	1
	Moderate	1,339,692	11.13	20	5.79	8
	High	1,344,707	11.17	61	17.36	25
	Very high	2,263,507	18.80	263	75.24	65
DLNN-P	Very low	5,609,514	46.59	8	2.25	1
	Low	1,634,496	13.58	10	2.89	3
	Moderate	1,258,738	10.46	27	7.72	12
	High	1,399,513	11.62	55	15.76	21
	Very high	2,137,060	17.75	250	71.38	63
DLNN-S	Very low	5,173,934	42.98	5	1.29	0
	Low	1,651,690	13.72	8	2.25	2
	Moderate	1,929,658	16.03	32	9.00	8
	High	1,549,959	12.87	98	27.97	31
	Very high	1,734,078	14.40	208	59.49	59
DLNN-RLV	Very low	5,286,053	43.91	5	1.29	0
	Low	2,235,567	18.57	9	2.57	2
	Moderate	1,304,946	10.84	25	7.07	9
	High	1,448,229	12.03	102	29.26	33
	Very high	1,764,525	14.66	209	59.81	56

### Factors importance analysis

**Figure 12:**
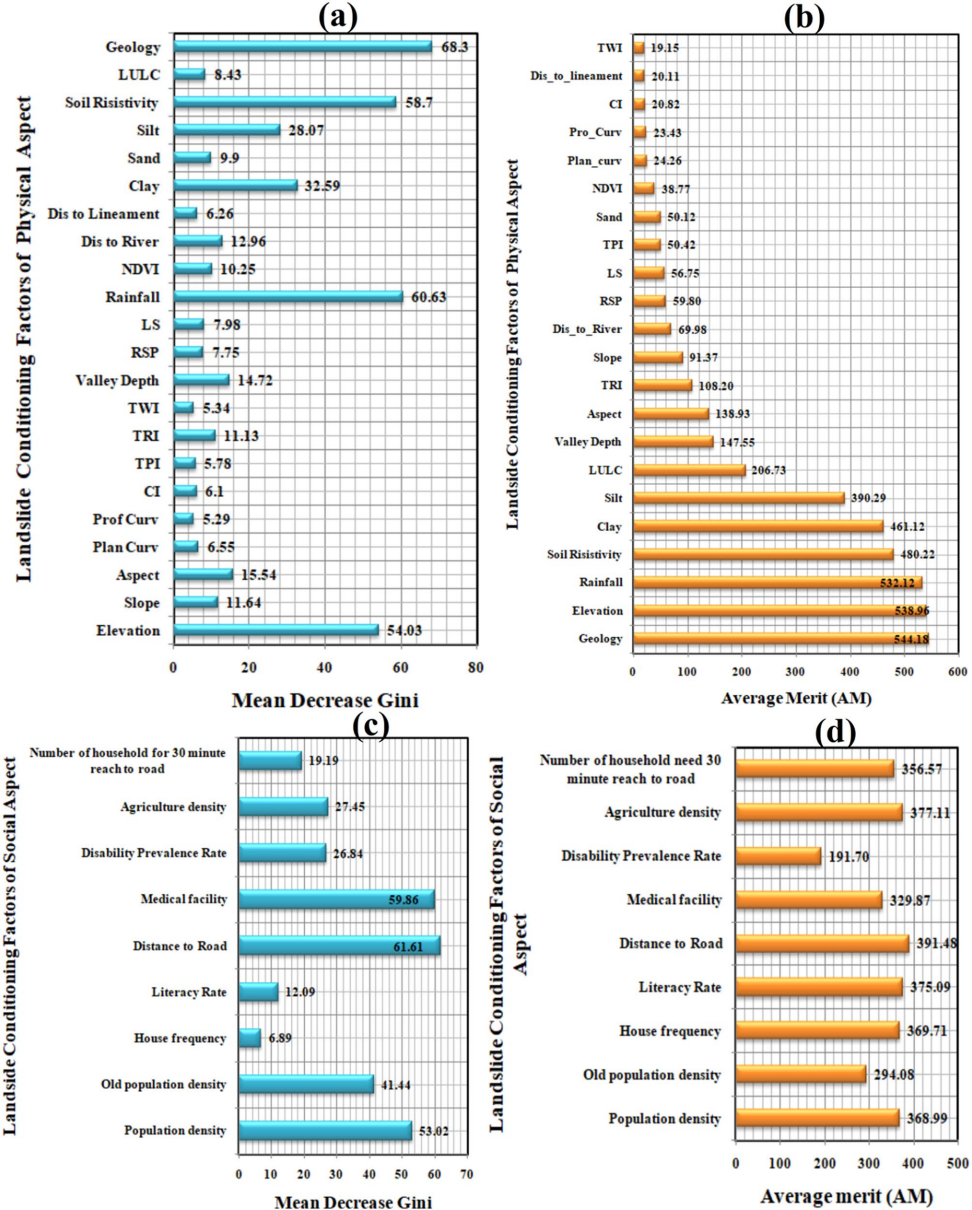
Importance of the LVCFs: (**a**) mean decrease Gini of geo-environmental LVCFs by RF, (**b**) average merit of geo-environmental LVCFs by CSAE, (**c**) mean decrease Gini of socio-economic LVCFs by RF, (**d**) average merit of socio-economic LVCFs by CSAE.

The importance of the LVCFs in predicting landslide vulnerability was evaluated by random forest (RF) and chi-square attribute evolution technique (CSAE). The outcome of RF showed that the geology, rainfall, soil resistivity, and elevation were the most predictive factors for landslide vulnerability modelling in this research, followed by the sand and silt distribution and other geo-environment LVCFs (Fig. 12a). Among the socio-economic LVCFs, distance to road, population density factors have the most importance (Fig. 12c). The CSAE method yielded similar results to the RF model (Fig. 12b,d).

## Discussions

Identifying or mapping the areas with future landslide potential is one of the most useful document for appropriate land use planning and mitigation decision making^13,47,48^. Evaluation of landslide susceptibility and vulnerability of a hilly area (as mountainous areas are subjected to the landslides) is indeed, necessary as it will serve as an essential dataset which will be used for identifying the points and places of relatively high landslide susceptibility and vulnerability which can be utilized for efficient and safe planning, design as well as the construction activities in an identified landslide zone. In the studied region, as well as other similar locations, there has been a significant growth in human socio-economic activities as well as other geo-environmental variables in recent decades. As a result, the frequency with which landslides occur in certain locations has risen. Such occurrences result in a significant loss of human life and property, as well as it results in a negative impact on different sectors such as tourism and infrastructure development^49^. A better working LVM has always been a subject of significant relevance within the community of numerous scholars across the world who are researching and investigating landslides. This is due to the fact that the methodology and conditioning factors used can have a substantial impact on the models' predictive performance^48^. It is very relevant to employ the mechanisms of factor selection to maximize the efficiency of landslide models by eliminating unwanted or trivial variables before training those^50^. CA and IGR method was adopted to accomplish this. In this study, CA revealed that all of the conditioning variables were effective and independent. In order to pick the most appropriate conditioning factor, there must be a non-collinear link between the LVCFs, which may be done via multicollinearity analysis^51^. Applying CA and IGR analysis, this study took into account a total of twenty-two geo-environmental and nine socio-economic factors, and the incorporation of these LVCFs has been justified in several studies^4,5,21^. It is very important to select LVCFs associated with past and present landslide events in vulnerability modelling. Although landslides are quasi-natural hazard but when it results in destruction and harm to the economy and society, it indicates the association of socio-economic causes in the event happened. Therefore, to enhance the performance of modelling, this research was conducted taking into consideration both physical and social aspects; these factors are found to be very significant, especially in hillslope regions^52^.

The study was approached towards mapping the landslide vulnerability considering geo-environmental and socio-economic aspects to improve the classification accuracy using deep learning and benchmark machine learning methods viz. CNN, DLNN and ANN. In all cases of vulnerability mapping (Physical, socio-economic and combined) opted in this study, CNN has shown the best result followed by DLNN and ANN. In terms of the validation measures, the CNN-RLV model had the highest goodness-off-fit and excellent predictive performance, followed by the CNN-LS, CNN-SV, DLNN-LS, DLNN-S, DLNN- RLV, ANN-LS, ANN-SV and ANN-RLV models. Sadighi et al.^53^ for landslide susceptibility assessment used MLP-NN with a Back-Propagation algorithm (BPANN), Adaptive Neuro-Fuzzy Inference System (ANFIS) models. However, result of models shows that the ANFIS-ICA had the superior results but ANN had quite good predictive accuracy i.e. AUC of 88.8%. In this study, ANN-RLV model has assured 88.7% success rate by the application of the AUC measure which is similar to the aforementioned study. In landslide susceptibility assessments, Bui et al.^9^ used a DLNN model and compared its predictive efficiency with state-of-the-art machine learning models in Kon Tum province, Vietnam. Using ROC curve, the performance of the models revealed that the DLNN model had the highest goodness-of-fit and outperforming ANN, SVM model. Relatively better performance of DLNN than of ANN was also achieved in landslide vulnerability mapping for the present research. The efficiency of deep learning models compared to ML models was found to be better as per the study of Yao et al.^54^ where authors have developed the deep neural network model based on semi-supervised analysis (SSL-DNN) for the landslide susceptibility estimation. For comparison, supervised models were introduced, including deep neural network (DNN), SVM, and logistic regression (LR). The result revealed that all comparable models were surpassed by the proposed SSL-DNN (AUC = 0.898) which is greatly supportive of outcome of the deliberating research. Application and enhanced competence of DLNN model can also be found in other hazard vulnerability and susceptibility mapping as Band et al.^55^ proposed a DLNN model and an ensemble particle swarm optimization (PSO) algorithm with DLNN (PSO-DLNN), for gully erosion susceptibility mapping. These models were compared with ANN and SVM model. The PSO-DLNN model has the highest efficiency followed by the ANN and SVM. Therefore, the derived outcome of this research has a similar covenant to the very previous studies delegating relevance of adopted methodological scheme. Above all, r, the convolution neural network (CNN) model has provided superior results outperforming DLNN and ANN models in all of the social vulnerability, landslide susceptibility and relative or combined vulnerability assessment as exposed by all the adopted validation measures both in training set and testing set based analysis (Table 4). Therefore, accuracy of the DLNN model was better than the conventional ANN machine learning technique. This is because greater number of samples and huge data could be handled by this model and the outcomes can be estimated with greater precision. The key benefit of deep learning is its formal system of self-governing DLNN layer organization learning. The conventional machine learning is incapable of processing such a vast number of inputs, and the result is less optimal in comparison to the deep learning model^55^. The accuracy of machine learning for different purposes was substantially enhanced in deep learning systems^55^. Yi et al.^50^ developed a convolutional neural network (CNN) model for the spatial prediction of landslides. The result of CNN was compared with three conventional ML algorithms, i.e., logistic regression, multilayer perceptron (MLP) neural network and radial basis function (RBF) neural network which found CNN as the best fitted and excellent predictive model, followed by the MLP, logistic regression RBF. Wang et al.^39^ applied the deep learning and ML models such as logistic regression, SVM, RF models for LS assessment. The result of that research also proved that CNN had the highest performance of predictive modelling followed by the ML models. With the agreement of the results of these studies, the present work also confirms the relatively highest adaptability of CNN model in deriving LVMs as reflected in the produced results of validation and accuracy measures. ReLU activation function was applied in the present study considering the aforesaid literature. The advantages of CNN model are that it considers all the neighbourhood information and can determine manifold stages of representations from input data^56^^.^ It maintains the association of pixels using several factors and identifying internal elements^59^.

Following Jenk’s algorithm of natural breaks classification, LVMs were divided into five groups of susceptibility classes. This strategy of clustering data helps to reduce the mean–variance of each class from the mean within class range and to increase the discrepancy between each class from the means of the other classes^57^. From the analysis of the LVMs of this study, the very high vulnerability zone of landslide is found in the southern, south-western and south-eastern portion where the soil resistivity and geology is very weak. The amount of annual average precipitation is maximum in this part of the district. With very high elevation and steep slope, certain geological configurations influenced by socio-economic aspects tend to become unstable causing landslide. Phuentsholing is a highly urbanized centre located in the south upland slope with dense road network built through modification of the general slope that accelerates landslide processes.

Landslides are caused by a number of factors in a given area, but not all of them are equally responsible. During the field investigation, it was found that the landslides in the study region are caused by both natural causes (geological structure, heavy rainfall and very steep slope) as well as by human interferences (such as slope cutting for the construction of road, deforestation for expansion of agricultural land) which makes the area vulnerable to landslide. Relative landslide density index (R), in this work, helped to analyse the association between produced vulnerability classes of landslide models and percentage of inventory landslides. The highest R-index value can be found in very high vulnerability class of each model followed by high vulnerability class which is positive for validating models.

The factor importance using RF (mean decrease Gini) and CSAE (average merit) represented the most important predictive LVCFs which are fittingly prominent in the southern part of the district (Fig. 12). Among the physical conditioning factors, both the RF and CSAE based evaluation has identified elevation, rainfall, geology, soil resistivity, soil clay and silt percentage etc. as the foremost persuasive factors for land sliding. The alike importance of the factors has reflected in several pieces of research such as elevation has been identified as a critical LVCF in various literature since most landslides occur in mountainous regions with a specific gradient^4,7,10^. Landslides are influenced by the structure, ordination, age, and exposure of the underlying surface^52^. The soil resistivity parameter has a positive connection with landslide susceptibility, showing that reducing soil resistance upturns the chance of a landslide, especially at higher elevations. Increased soil clay and silt content at medium to high altitude and upslope areas remain more unstable due to lower integrity than rocky sections, making it more vulnerable to seepage erosion, liquefaction, and fluidization. However, the type of soil and the amount of vegetation cover plays an important influence in this^46^. Among the socio-economic factors, RF and CSAE method have recognised distance to road, population density, agricultural density, house frequency factor as crucial for making the area landslide vulnerable. A negative correlation exists for the distance to road LVF, indicating that the severity of possible landslide events increases in places as roads get closer, and vice versa. A substantial amount of work by Chan et al.^58^, Weigand et al.^59^ has established the role of these factors in triggering landslides.

## Conclusion

Landslides have been seen in recent decades as the most critical natural risk that poses serious threat to both life and property all over the world. Thus short-term and long-term solutions are considered necessary to confront these daunting challenges. The landslide vulnerability map has recently become an important means of delineating landslide-prone regions and management. With the aid of sophisticated methods, proper data, and integration of remote sensing and a geographical information system, this can be accomplished. DLNN, ANN, and convolution neural network (CNN) models were used and r all aspects of a landslide event were considered which are novel approaches to perform the landslide vulnerability mapping of the district. Therefore, along with geo-environmental data, potential socio-economic factors were also considered as LVCFs using advanced factor selection techniques. CNN model achieved highest accuracy in modelling the vulnerability in the study area. As per the finding of the models, lower part of the district is highly susceptible and it needs immediate measures for managing. For the landslide risk supervision in the present and also for the future, the LVM can suggest implementing different management strategies like afforestation, barrier construction, and proper land use planning.
